# Robustness in jasmonate signaling: mechanisms of concerted regulation and implications for crop improvement

**DOI:** 10.1007/s42994-025-00244-1

**Published:** 2025-09-29

**Authors:** Ke Zhou, Tiantian Han, Bingqing Pan, Xiaomeng Hu, Xiaomei Chen, Xinyu Liu, Shihong Fei, Yating Yang, Wenhao Li, Minmin Du

**Affiliations:** 1 Beijing Key Laboratory of Growth and Developmental Regulation for Protected Vegetable Crops, College of Horticulture, China Agricultural University, Beijing, 100193 China; 2 Sanya Institute of China Agricultural University, Sanya, 572025 China; 3 Yazhouwan National Laboratory, Sanya, 572024 China; 4 Sichuan Agricultural University, Chengdu, 625014 China

**Keywords:** Jasmonic acid, Transcriptional regulation, JAZ, COI1, MYC2, Growth, Defense, Feedback regulatory mechanisms

## Abstract

The jasmonate signaling pathway coordinates plant defenses and growth, thereby enhancing fitness in changing conditions. Jasmonate-mediated responses are triggered by the recognition of external signals via pattern recognition receptors (PRRs) located on the cell membrane. Following signal perception, cells rapidly activate jasmonic acid (JA) biosynthesis, resulting in the accumulation of the bioactive jasmonate, jasmonoyl-isoleucine (JA-Ile). In the nucleus, the coronatine insensitive 1–jasmonate-ZIM-domain (COI1–JAZ) complex recognizes JA-Ile and triggers JAZ ubiquitination and proteasomal degradation. Consequently, transcription factors (e.g., MYC2) bound by JAZ are released, enabling the activation and amplification of JA responses. In parallel to this activation, feedback regulation orchestrated by transcription factors terminates transcription, preventing overcommitment to JA signaling. In this review, we summarize recent advances in understanding JA signaling, emphasizing the connection between PRR activation and JA biosynthesis, and the feedback regulatory mechanisms that ensure precision and robustness of the JA signaling pathway. Finally, we discuss how these mechanistic insights can be leveraged to optimize JA signaling for crop genetic improvement.

## Introduction

Plants balance growth and defense in response to fluctuating conditions (Guo et al. 2018a; He et al. 2022; Züst and Agrawal 2017). For example, jasmonate signaling pathways coordinate stress tolerance and growth responses, enabling plants to maintain their fitness under uncertain conditions. Jasmonates (JAs), including jasmonic acid (JA) and its derivatives, are ubiquitous lipid-derived phytohormones that regulate stress response as well as plant growth and development. The isolation and purification of methyl jasmonate (MeJA) from the flowers of Royal Jasmine (*Jasminum grandiflorum*) was first reported in 1962 (Demole et al. 1962). In 1980, the Japanese scientist Ueda and his colleagues discovered that JA accelerates plant senescence (Ueda and Kato 1980). A landmark study from the Ryan laboratory in 1990 demonstrated that MeJA induces defense-related gene expression in leaves, providing the first clear evidence that jasmonates function in plant disease resistance (Farmer and Ryan 1990). Subsequent studies have firmly established the JA signaling pathway at the core of plant defenses, where it acts in initiating resistance against chewing insects, mounting a response to mechanical injury, and defending against necrotrophic pathogens (Howe et al. 2018; Li et al. 2023; Zhang et al. 2017). JA signaling also affects plant growth and development, where it acts in regulating the development of flowers, gametes, and trichomes, as well as sex determination (Huang et al. 2023a, 2017b). In addition, JA affects plant acclimation to abiotic stresses, such as high salinity, drought, and extreme heat or cold (Hu et al. 2013b; Waadt et al. 2022; Wang et al. 2021).

**Fig. 1:**
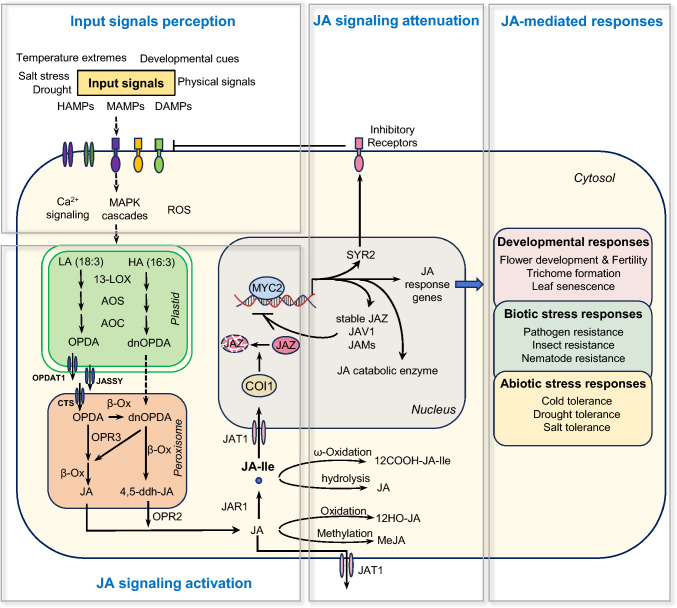
Overview of jasmonate-mediated stress and developmental responses. Pattern recognition receptors (PRRs) on the cell surface recognize input signals from plant interactions with their environment (biotic/abiotic) or developmental processes. PRR activation initiates intracellular signaling cascades, including mitogen-activated protein kinase (MAPK) signaling, calcium signaling, and reactive oxygen species (ROS) production, as well as other pathways. However, it is still unclear how these signaling events connect to the induction of jasmonic acid (JA) signal. The enzymatic conversion of linolenic acid (LA) to JA occurs in plastids and peroxisomes. The resulting JA is transported to the cytoplasm, where it is conjugated to form the active signal molecule JA-Ile. In the nucleus, JA-Ile promotes COI1–JAZ complex formation, triggering JAZ ubiquitination and proteasomal degradation. JAZ degradation liberates transcription factors like MYC2 from repression, enabling the expression of JA-responsive genes crucial for development and stress adaptation. Furthermore, negative regulators induced by MYC2, such as the inhibitory receptor SYR2, transcription factor JAM, JA-Ile catabolic enzymes, and newly synthesized JAZ proteins, act to dampen JA signaling intensity. Abbreviations: Microbe-associated molecular patterns (MAMPs), damage-associated molecular patterns (DAMPs), herbivore-associated molecular patterns (HAMPs), mitogen-activated protein kinase (MAPK), linolenic acid (LA), hexadecatrienoic acid (HA), lipoxygenase (LOX), allene oxide synthase (AOS), allene oxide cyclase (AOC), 2,3-dinor-12-oxo-10,15(Z)-phytodienoic acid (dnOPDA), 4,5-didehydro-JA (4,5-ddh-JA), OPDA reductase 3 (OPR3), OPDA reductase 2 (OPR2), jasmonate resistant 1 (JAR1), 12-oxo-phytodienoic acid (OPDA), β-oxidation (β-ox), jasmonoyl-L-isoleucine (JA-Ile), OPDA transporter 1 (OPDAT1), COMATOSE (CTS), jasmonate transporter 1 (JAT1), systemin receptor 2 (SYR2), coronatine insensitive 1 (COI1), jasmonate-associated VQ-motif gene 1 (JAV1), jasmonate-ZIM domain (JAZ), jasmonate-associated MYC2-like (JAM), 12-carboxy-JA-Ile (12COOH-JA-Ile), 12-hydroxy-JA-Ile (12OH-JA-Ile), methyl jasmonate (MeJA)

Plant–environment and plant–pathogen interactions or developmental cues rapidly induce JA biosynthesis (Gasperini and Howe 2024; Howe et al. 2018). For example, recognition of pathogen-related molecules by cell-surface receptors triggers a signal transduction cascade that rapidly induces de novo lipid biosynthesis in chloroplasts and leads to the production of jasmonoyl-isoleucine (JA-Ile). This bioactive form JA-Ile subsequently activates the JA signaling pathway in the nucleus to modulate plant immunity, development, and growth. Furthermore, once the JA signaling pathway is activated, plants initiate feedback regulatory mechanisms that ensure JA homeostasis. This regulatory mechanism fine-tunes the signaling network, allowing plants to balance growth and defense and adjust to fluctuating conditions (Fig. 1).

Many excellent reviews have summarized JA biosynthesis, JA signaling, and JA functions in regulating plant defenses against abiotic and biotic stresses (Gasperini and Howe 2024; Howe et al. 2018; Huang et al. 2023b; Li et al. 2023; Sun et al. 2011; Wasternack and Hause 2013; Zhang et al. 2017). This review highlights recent advances in JA research, emphasizing how the activation of pattern recognition receptors (PRRs) is linked to JA biosynthesis and how transcriptional regulation maintains the specificity and robustness of this pathway. In addition, this review discusses how recent advances in JA signaling research can be integrated with biotechnological strategies to accelerate genetic improvement in crops. Drawing on findings from the JA signaling pathway, this review aims to establish a theoretical framework for studying other signaling pathways and to provide insights into the genetic improvement of crops to address global climate challenges and promote sustainable agriculture.

## Activation of the JA signaling pathway

### Triggering JA biosynthesis

Plants sense developmental cues and stress signals from attacking organisms, damaged cells, and abiotic factors, including damage-associated molecular patterns (DAMPs), microbe-associated molecular patterns (MAMPs), herbivore-associated molecular patterns (HAMPs), and physical signals, such as changes in electrical signals or turgor pressure (Farmer et al. 2014; Johns et al. 2021; Ngou et al. 2022; Reymond 2021). Upon recognition by PRRs, these signals induce the rapid accumulation of JA, which will activate the JA signaling pathway (Fig. 1) (Erb et al. 2012; Glauser et al. 2008; Koo et al. 2009). Notably, expression of JA-responsive genes in response to wounding occurs in the damaged (local) tissues and in distant (systemic) tissues that have not been damaged, a phenomenon known as systemic wound response (Green and Ryan 1972; Ryan 1990). Among well-characterized DAMPs, peptide phytohormones (systemin in tomato [*Solanum lycopersicum*], AtPep1 in Arabidopsis [*Arabidopsis thaliana*], and ZmPep3 in maize [*Zea mays*]), oligogalacturonides, and extracellular ATP promote rapid biosynthesis of JA and trigger JA-mediated defense responses (Gust et al. 2017). In addition, certain herbivore-derived HAMPs, such as fatty acid–amino acid conjugates (FACs), induce JA responses through mechanisms that remain to be elucidated (Snoeck et al. 2022). Wound-induced electrical signals also rapidly trigger de novo JA biosynthesis and act as systemic signals that propagate defense responses to undamaged tissues; this propagation requires members of the glutamate receptor-like (GLR) protein family (Johns et al. 2021). Nevertheless, the mechanisms by which these signaling pathways regulate JA biosynthesis remain largely unknown.

Systemin in tomato is a well-known upstream signal in JA signaling. Systemin is the first peptide hormone identified in plants and is a biologically active 18-amino acid peptide derived from the proteolytic cleavage of the C-terminal region of its precursor peptide prosystemin (Pearce et al. 1991). Systemin application or *PROSYSTEMIN* overexpression in tomato strongly activated the expression of JA-mediated defense-related genes (McGurl et al. 1994; Pearce et al. 1991). A genetic screen in tomato for *suppressor of prosystemin-mediated response* (*spr*) mutants identified key components of the JA biosynthesis and signaling pathways, supporting the idea that systemin and JA regulate plant defenses by a common signaling pathway (Howe and Ryan 1999; Sun et al. 2011; Yan et al. 2013b; Zhou et al. 2025). Although systemin and JA were once considered as candidates to be the systemic signal, grafting experiments with JA biosynthesis and signaling mutants showed that JA or its derivatives, and not systemin, are the systemic signals (Li et al. 2002). After decades of searching, two independent research groups have successfully cloned the systemin receptor using forward genetics (Wang et al. 2018; Zhou et al. 2025). *Systemin receptor 1* (*SYR1*, also reported as *SUPPRESSOR OF PROSYSTEMIN-MEDIATED RESPONSES 1* [*SPR1*]) encodes a leucine-rich repeat (LRR) receptor-like kinase that is a genuine receptor for systemin (Wang et al. 2018; Zhou et al. 2025). *spr1* mutants exhibited weakened local wound responses and nearly abolished systemic wound responses, resembling those of *prosystemin* mutants (Lee and Howe 2003; Zhou et al. 2025). These findings indicate that wounding triggers systemin accumulation, which amplifies local JA signaling, promotes long-distance jasmonate transport, and activates systemic responses. Further research is warranted to uncover the precise molecular mechanisms linking systemin signaling to JA signaling.

When PRRs at the plant cell-surface sense danger or developmental signals, they initiate a series of intracellular signaling cascades. Similar to systemin-triggered signaling, these cascades involve plasma membrane depolarization, a rise in intracellular Ca^2+^ concentrations, lower H^+^-ATPase activity, a reactive oxygen species (ROS) burst, initiation of mitogen-activated protein kinase (MAPK) cascades, activation of phospholipases, and release of α-linolenic acid (Ryan 2000; Sun et al. 2011; Zhang et al. 2020b). Together, these processes drive the rapid accumulation of JA in plants.

Ca^2+^ serves as a key second messenger in animals and plants. In plants, transient elevations in cytosolic Ca^2+^ levels, known as Ca^2+^ waves, are induced by various stimuli, including herbivory attack, mechanical wounding, and exposure to molecular patterns (HAMPs, DAMPs, and MAMPs) (Choi et al. 2016; Gilroy et al. 2016). In Arabidopsis, the development of fluorescent biosensors for cytosolic Ca^2+^ allowed real-time visualization of systemic Ca^2+^ wave propagation triggered by insect feeding or mechanical wounding (Nguyen et al. 2018; Toyota et al. 2018; Yan et al. 2018a). Pretreatment of wounded petioles with the Ca^2+^ channel inhibitor lanthanum (La^3+^) effectively blocked the Ca^2+^ waves, resulting in diminished induction of JA-responsive gene expression in systemic leaves; these results show the role of Ca^2+^ signaling in activation of systemic JA signaling following wounding (Toyota et al. 2018; Yan et al. 2018a). Furthermore, systemic propagation of wound-induced Ca^2+^ waves, much like electrical signals, relies on clade III GLR family members, which regulate the expression of JA-responsive genes (Toyota et al. 2018). Elevated cytosolic Ca^2+^ levels trigger calmodulin-dependent jasmonate-associated VQ-motif gene 1 (JAV1) phosphorylation, which disrupts the JAV1–jasmonate-ZIM-domain 8 (JAZ8)–WRKY51 (JJW) complex and relieves its repression of JA biosynthetic gene expression, thus inducing JA accumulation (Yan et al. 2018a).

Wounding or tissue damage caused by herbivory triggers rapid ROS accumulation and the propagation of ROS waves that induce systemic wound responses (Fichman and Mittler 2020, 2021; Malook et al. 2021; Mittler et al. 2022; Wang et al. 2024). The NADPH oxidase RESPIRATORY BURST OXIDASE HOMOLOG D (RBOHD) mediates ROS production and ROS wave propagation, and its activity is closely associated with local and systemic JA biosynthesis (Miller et al. 2009; Orozco-Cárdenas et al. 2001; Zandalinas and Mittler 2021). Moreover, RBOHD activity is regulated by Ca^2+^ binding and post-translational modifications such as phosphorylation. Intracellular Ca^2+^ can directly bind to the EF-hand domain of RBOHD, or indirectly activate calcium-dependent protein kinases to modulate its activity (Gao et al. 2013; Ogasawara et al. 2008). The receptor-like cytoplasmic kinase BIK1, a hub of PRR-mediated signaling cascades, regulates RBOHD both directly through phosphorylation and indirectly via modulation of MAMP-triggered Ca^2+^ influx, thereby providing a mechanistic link between MAMP perception and Ca^2+^-dependent immune regulation (Kadota et al. 2014; Li et al. 2014).

MAPK cascades mediate signal transduction downstream of PRRs activation (Campos et al. 2014; Zhang and Zhang 2022). Silencing specific *MAPK* genes suppresses JA biosynthesis and downregulates JA-responsive gene expression, consistent with their role in the early activation of JA biosynthesis (Kandoth et al. 2007; Wu et al. 2007). For example, silencing tomato *LeMPK1*, *LeMPK2*, and *LeMPK3* attenuated JA biosynthesis and compromised plant resistance to larvae of the tobacco hornworm (*Manduca sexta*) (Kandoth et al. 2007). In addition, silencing *ZmMPK4* elevated JA and JA-Ile levels in maize upon wounding or insect attack, but diminished the resistance of maize plants to larvae of the oriental armyworm (*Mythimna separata*). Given the inconsistency with the well-established role of JA in insect resistance, further investigation is needed to clarify the function of ZmMPK4 (Li et al. 2024b; Zhang et al. 2021).

These early events, including Ca^2+^ signaling, the ROS burst, and MAPK cascades, and their crosstalk, are critical for the activation of JA biosynthesis. A key challenge in this field will be to establish direct molecular links between the perception of signals (e.g., MAMPs, HAMPs, DAMPs, and electrical signals) and JA biosynthesis through ROS, Ca^2+^ signaling, and MAPK signaling cascades.

### JA biosynthesis, metabolism, and transport

JA biosynthesis begins with its precursor, α-linolenic acid (ALA, 18:3), via octadecanoid pathway enzymes that localize in the chloroplast, peroxisome, and cytoplasm (Fig. 1) (Chini et al. 2018; Wasternack and Hause 2013, 2018). This pathway can be categorized into three steps based on the subcellular localization of these enzymes. In the first step, within the chloroplast, phospholipase A1 (PLA1) releases ALA from plastid membrane lipids, and 13-lipoxygenase (13-LOX), allene oxide synthase (AOS), and allene oxide cyclase (AOC) convert ALA into 12-oxo-phytodienoic acid (OPDA). In the second step, OPDA is transported to the peroxisome, where it is converted to ( +)-7-iso-JA by OPDA reductase 3 (OPR3), OPC-8 CoA ligase (OPCL), acyl-CoA oxidase (ACX), multifunctional protein (MPF), and 3-keto-acyl-CoA thiolase (KAT). In the final step, the cytoplasmic JA-Ile synthetase jasmonate resistant 1 (JAR1) conjugates JA and isoleucine (Ile), to produce the bioactive compound ( +)-7-iso-JA-Ile (Chini et al. 2018; Wasternack and Hause 2013, 2018; Staswick and Tiryaki 2004).

An alternative OPR3-independent JA biosynthesis pathway has been identified in Arabidopsis, involving the conversion of hexadecatrienoic acid (16:3) into 2,3-dinor-12-oxo-10,15-(*Z*)-phytodienoic acid (dnOPDA) in the chloroplast, followed by dnOPDA transport to the peroxisome. In the peroxisome, OPDA undergoes a single round of β-oxidation to form dnOPDA, which undergoes two additional rounds of β-oxidation to produce 4,5-didehydro-JA (4,5-ddh-JA); OPR2 reduces 4,5-ddh-JA in the cytoplasm to yield JA (Chini et al. 2018).

JA homeostasis and signaling also involve JA metabolism (Fig. 1) in which is catabolized to bioactive or inactive products. For example, in the cytoplasm, JAR1 converts JA to the primary bioactive form, JA-Ile. In addition, bioactive conjugates of JA to other amino acids, including alanine, valine, leucine, and methionine, may act through signaling pathways dependent on the JA receptor CORONATINE INSENSITIVE 1 (COI1) (Yan et al. 2016). JA also undergoes modifications, such as hydroxylation, glycosylation, and methyl esterification (Wasternack and Feussner 2018; Wasternack and Hause 2013). JA is converted into the volatile MeJA via the action of JA CARBOXYL METHYLTRANSFERASE (JMT), a modification that can be reversed by MeJA ESTERASE (MJE) (Wasternack and Hause 2013). JA can also be directly converted into 12-hydroxy-JA (12-OH-JA) by jasmonate-induced oxygenases (JOXs) or jasmonic acid oxidases (JAOs) (Caarls et al. 2017; Smirnova et al. 2017). 12-OH-JA can be further metabolized into sulfate derivatives (12HSO_4_-JA) or glucose conjugates (12-*O*-Glc-JA). The pentenyl side chain of JA-Ile undergoes successive oxidation by the cytochrome P450 (CYP) 94 family members (CYP94B1, CYP94B3, and CYP94C1), producing 12-OH-JA-Ile and 12-COOH-JA-Ile (Heitz et al. 2012; Koo et al. 2011, 2014). In addition, JA-Ile or 12-OH-JA-Ile can be converted into JA or 12-OH-JA by amidohydrolases (IAA-alanine resistant 3 [IAR3] and AA-leucine resistant-like gene 6 [ILL6]) (Bhosale et al. 2013; Widemann et al. 2013; Zhang et al. 2015).

JA biosynthesis, metabolism, and signaling all involve jasmonates transport. By modulating JA levels in cells or intracellular compartments, jasmonates transporters fine-tune various aspects of plant physiology (Fig. 1) (Li et al. 2021a; Zhang et al. 2023b). For example, the OPDA TRANSPORTER 1 (OPDAT1) and JASSY channels are located in the inner and outer envelope of chloroplast, respectively, and they may function cooperatively to efflux OPDA into the cytoplasm (Guan et al. 2019; Zhao et al. 2021). The ATP-binding cassette (ABC) transporter COMATOSE (CTS), located in the peroxisomal membrane, mediates OPDA influx into the peroxisome (Theodoulou et al. 2005). In Arabidopsis, the jasmonate transporter (JAT) family, a group of half-size ATP-binding cassette G (ABCG) transporters, has been reported to be involved in jasmonate transport (Li et al. 2020, 2017; Wang et al. 2019a). JAT1 (also reported as ABCG16) localizes to the nuclear membrane, where it mediates JA-Ile influx and to the plasma membrane, where it mediates JA efflux (An et al. 2024; Li et al. 2017). *jat1* mutants exhibited lower expression of JA-responsive genes, less-pronounced inhibition of root growth in response to JA treatment, and larger organs (including leaves, flowers, and seeds), phenotypes that are reminiscent of *coi1* mutants (Li et al. 2017). Recently, a structural analysis revealed that JAT1 recognizes and transports JA, but not abscisic acid (ABA) (An et al. 2024). JAT2, located at the peroxisomal membrane, may function in JA export from the peroxisome (Wang et al. 2019a). Furthermore, JAT3 and JAT4, located at the plasma membrane, mediate the influx of JA and are essential for systemic wound responses (Li et al. 2020). The *jat3 jat4* double mutant was defective in JA translocation, resulting in a severe weakening or complete loss of systemic wound responses (Li et al. 2020). Although several jasmonate transporters have been identified, their functional characterization has largely depended on in vitro systems, and direct evidence for their transport activity *in planta* is still lacking. Furthermore, the key transporter mediating long-distance JA translocation remains unknown, underscoring the need for broader efforts to identify additional jasmonate transporters.

### JA perception and signal transduction

JA perception is a critical step in the JA signaling pathway. Identification of the JA receptors and elucidation of their action mechanisms mainly rely on genetic and biochemical studies. In 1994, Feys et al. identified the Arabidopsis *coronatine insensitive1* (*coi1*) mutant, which does not respond to the JA analog coronatine (COR) and to MeJA, in addition to being male sterile (Feys et al. 1994). *COI1* encodes an F-box protein containing an LRR domain (Xie et al. 1998). Subsequent biochemical assays showed that in Arabidopsis, COI1 forms an SCF^COI1^ E3 ubiquitin ligase complex with SKP1-like 1 (ASK1) or ASK2, ring-box 1 (RBX1), and cullin 1 (CUL1) (Devoto et al. 2002; Xu et al. 2002). Moreover, its interaction with ASK1 and CUL1 stabilizes COI1, indicating that the SCF^COI1^ complex maintains COI1 stability (Song et al. 2021; Yan et al. 2013a). In agreement with this notion, loss-of-function mutants in components of the SCF^COI1^ complex *in planta*, such as *ASK1* and *CUL1*, resulted in lower COI1 protein levels and defective JA responses (Xu et al. 2002; Yan et al. 2013a). Notably, COI1 is homologous to the auxin receptor TRANSPORT INHIBITOR RESPONSE 1 (TIR1), another F-box protein. Based on the crystal structure of TIR1, the LRR domain of COI1 is thought to participate in JA-Ile binding and recognition. Further biochemical experiments confirmed that COI1 directly binds to JA-Ile and COR, establishing it as a receptor for JA-Ile (Thines et al. 2007; Yan et al. 2009).

Many dicots have a single *COI1* gene, whereas some monocots have multiple copies. For example, *Arabidopsis* and tomato have single *COI1* homologs, but rice (*Oryza sativa*) and maize have three and six *COI1* homologs, respectively (Lee et al. 2013; Qi et al. 2022b). In tomato, the *jasmonic acid insensitive1* (*jai1*) mutant, which harbors a mutation in the Arabidopsis *COI1* homolog, exhibits impaired JA-induced defense responses, defective glandular trichome development, female sterility, and lower pollen viability (Dobritzsch et al. 2015; Li et al. 2004). Heterologous expression of maize *ZmCOI1a*, *ZmCOI1b*, or *ZmCOI1c*, but not *ZmCOI2*, in the Arabidopsis *coi1-1* mutant can restore male fertility and disease resistance (An et al. 2019). However, the *Zmcoi2a Zmcoi2b* double mutant has defects in anther dehiscence and pollen germination (Qi et al. 2022b). In rice, OsCOI1b regulates the effects of JA on root growth and grain size, and functions redundantly with OsCOI1a to regulate the development of spikelets. OsCOI2 controls male fertility, along with the effect of JA on root growth, leaf senescence, and grain size. Furthermore, OsCOI1a, OsCOI1b, and OsCOI2 promote resistance to the brown planthopper (*Nilaparvata lugens*) via distinct mechanisms (Wang et al. 2023).

The Arabidopsis *jasmonate-insensitive 1* (*jin1*) mutant  showed reduced JA-mediated root growth inhibition and diminished expression of  JA-responsive gene (Berger et al. 1996). *JIN1* encodes the basic helix–loop–helix (bHLH) transcription factor MYC2, a master transcription factor in the JA signaling pathway (Berger et al. 1996; Boter et al. 2004; Fernandez-Calvo et al. 2011; Kazan and Manners 2013; Lorenzo et al. 2004). MYC2 specifically binds to the G-box (CACGTG) and G-box-related (CACATG/CACGTT) sites in its target promoters to regulate gene expression (Dombrecht et al. 2007). Chromatin immunoprecipitation sequencing (ChIP-seq) and transcriptomic data from Arabidopsis, tomato, and rice show that MYC2 orchestrates JA-mediated transcriptional cascades by directly activating the gene expressio n of secondary transcription factor genes across different species. Thus, MYC-mediated transcriptional cascades activate and amplify the transcriptional reprogramming of the JA signaling pathway (Boter et al. 2004; Chen et al. 2024; Du et al. 2017; Ma et al. 2023).

In eukaryotes, the Mediator (MED) complex (Soutourina 2018; Zhai and Li 2019) connects transcription factors and RNA polymerase II (Pol II), promoting transcription initiation complex formation and activating the transcription of target genes (Soutourina 2018; Zhai et al. 2020). Arabidopsis MED25 was the first subunit of the Mediator complex that was shown to act in JA signaling (Chen et al. 2012). Loss of MED25 function leads to lower expression of JA-responsive genes and diminished resistance to necrotrophic pathogens (Chen et al. 2012). MYC2 interacts with MED25, and the resulting MYC2–MED25 complex (MMC) facilitates the JA-dependent recruitment of Pol II and other transcriptional co-factors to MYC2 target gene promoters (Zhai et al. 2020). This JA-dependent recruitment confers specificity to Pol II-mediated regulation of gene expression. MYC2 mediates the transcriptional reprogramming of defense-related genes while activating the expression of genes encoding negative regulators that terminate or attenuate JA-induced defense responses (Zhai et al. 2020), thereby enabling plants to balance growth and defense in challenging environments, as discussed in detail below.

Identification of the bioactive JA conjugate JA-Ile, the receptor COI1, and the core transcription factor MYC2 raises an important scientific question: how is MYC2 activity linked to JA-Ile and the receptor COI1? The structure and function of COI1 suggest that a transcriptional repressor is ubiquitylated by the SCF^COI1^ complex and degraded via the 26S proteasome. In 2007, three independent laboratories discovered that the SCF^COI1^ complex acts on JAZ family proteins and these transcriptional repressors negatively regulate the JA signaling pathway (Chini et al. 2007; Thines et al. 2007; Yan et al. 2007). JAZ proteins feature three domains: a conserved N-terminal domain, along with a central ZIM domain and a highly conserved Jas domain at the C-terminus (Pauwels and Goossens 2011). JAZ proteins interact with different factors through different domains to form distinct functional complexes in a JA-dependent manner. The Jas domain mediates the JAZ–COI1 and JAZ–MYC2 interactions (Sheard et al. 2010; Zhang et al. 2015). The ZIM domain facilitates JAZ homodimer and heterodimer formation and interacts with the adapter protein NINJA and polycomb group (PcG) proteins, which in turn recruit co-repressors to mediate transcriptional repression (Pauwels et al. 2010; Pauwels and Goossens 2011; Zander 2021).

Structural and pharmacological analysis from Sheard et al. indicated that the COI1–JAZ complex acts as the JA-Ile receptor (Sheard et al. 2010). Binding assays showed that the COI1–JAZ complex has a higher affinity for COR than COI1 alone, and that the co-factor phosphoinositide enhances the interaction between COI1–JAZ and JA-Ile (Sheard et al. 2010). In contrast, Yan and colleagues revealed that COI1 functions as the primary JA receptor, which initially forms a COI1–JA-Ile complex and then recruits JAZ proteins to mediate derepression of JA signaling (Yan et al. 2018b).

**Fig. 2:**
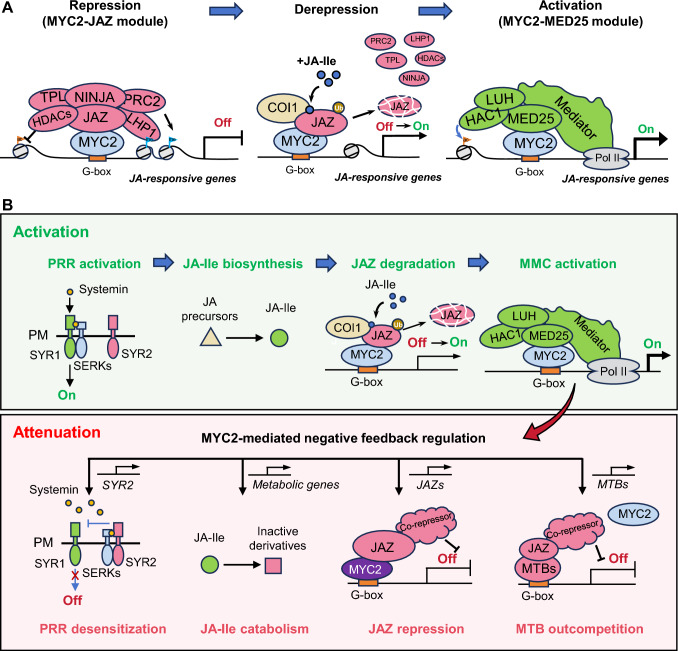
MYC2-mediated dynamic transcriptional regulation of the JA signaling pathway. **A** Activation of MYC2-mediated the JA signaling pathway. In the absence of bioactive jasmonate JA-Ile, JAZ proteins interact with MYC2 and are recruited to the promoter regions of MYC2 target genes, thereby inhibiting MYC2’s transcriptional activity through various adaptor proteins and co-repressors, including NINJA, TPL, and epigenetic-modifying enzymes. When JA-Ile is present, JA facilitates the interaction between COI1 and JAZ, leading to JAZ degradation via the ubiquitin–proteasome system. At the activation stage, JAZ degradation enhances the interaction between MYC2 and MED25, which in turn recruits RNA Polymerase II (Pol II) and coactivators (e.g., HAC1 and LUH) to the promoters of MYC2 target genes, thereby activating JA-responsive gene expression. **B** Feedback regulation of MYC2-mediated the JA signaling pathway. Many MYC2-targeted jasmonate-responsive genes encode components that attenuate the JA signaling pathway through negative feedback mechanisms. These include: the inhibitory receptor SYR2, which competes with SYR1 for binding to co-receptor SERKs in a ligand-concentration-dependent manner; JA-Ile catabolic enzymes, which turnover JA-Ile to attenuate JA responses; newly synthesized JAZ proteins, which restore the repression of target transcription factors; and JAM transcription factors, which compete with MYC2 for binding to G-box motifs and interact with JAZ to suppress JA-responsive gene expression. Abbreviations: TOPLESS (TPL), novel integrator of JAZ (NINJA), polycomb repressive complex 2 (PRC2), histone deacetylase (HDAC), like heterochromatin protein 1 LHP1, coronatine insensitive 1 (COI1), jasmonate-associated myc2-like (JAZ), RNA polymerase II (Pol II), systemin receptor 1 (SYR1) and SYR2, somatic embryogenesis receptor kinase (SERK), LEUNIG_HOMOLOG (LUH), histone acetyltransferase1 (HAC1), jasmonate-associated MYC2-like (JAM), plasma membrane (PM), ubiquitylation (Ub), H3K9ac (ac), H3K27me3 (me)

Beyond being JA-Ile co-receptors, JAZs inhibit the expression of JA-regulated genes through three main mechanisms (Fig. 2A): (1) without JA, the JAZ Jas domain binds to JA-related transcription factors, preventing them from recruiting the Mediator complex, and thus inhibiting their ability to activate transcription (Zhang et al. 2015); (2) JAZs also interact with NINJA, and the EAR transcriptional repression motif of NINJA or JAZ recruits the co-repressors TOPLESS (TPL) and TPL-related proteins to repress transcription (An et al. 2017; Pauwels et al. 2010; Zhai et al. 2020); and (3) JAZs also directly interact with the polycomb repressive complex 2 (PRC2) proteins, such as heterochromatin protein 1 (LHP1) and embryonic flower 2 (EMF2), to repress JA-responsive genes in Arabidopsis (Li et al. 2021b). Together, JAZ proteins act as transcriptional repressors that integrate signal perception with the regulation of JA-responsive gene transcription.

JA signaling pathway features a classic derepression model, analogous to that of the auxin signaling pathway (Fig. 2A). Specifically, under low JA-Ile concentrations, JAZ interacts with MYC2 and is recruited to the promoters of MYC2 target genes, thereby inhibiting MYC2 transcriptional activity. As plants respond to external stress, the concentration of JA-Ile rapidly rises. The COI1–JAZ receptor complex binds to JA-Ile, whereas MED25 facilitates the interaction between COI1 and JAZ, promoting the degradation of JAZ. That, in turn, enhances the interaction between MYC2 and MED25, facilitating the recruitment of Pol II and transcriptional co-factors to the promoters of MYC2 target genes. By linking MYC2 and Pol II, the Mediator complex promotes pre-initiation complex assembly and activates MYC2-mediated transcriptional reprogramming. Consequently, MYC2 exerts dual transcriptional regulatory roles within the JA-mediated transcriptional network via interactions with distinct co-factors. Specifically, when JA levels are low, MYC2 interacts with JAZs and transcriptionally represses target genes. In contrast, under high JA conditions, MYC2 interacts with MED25 to activate the transcription of target genes. Given that JA induces JAZ protein degradation via COI1, JA effectively converts MYC2 from a repressor (when bound to JAZ) to an activator (when bound to MED25) (Fig. 2A).

### JAZ–transcription factor modules regulate JA-mediated development and stress responses

In addition to MYC2, over 30 transcription factors (TFs) have been identified as direct JAZ interactors, regulating JA-mediated development and stress responses. Notably, JA serves a central role in defenses against necrotrophic pathogens, insect herbivory, and root-knot nematodes (RKNs) (Huang et al. 2023b; Wang et al. 2019b; Zhang et al. 2017). Group III bHLH TF family members are involved in the JA signaling pathway and function in the JAZ–TF interaction network in Arabidopsis (Goossens et al. 2017). Among them, members of the IIIe bHLH subclade, including MYC2, MYC3, MYC4, and MYC5, are key components of the best-characterized JAZ–TF modules involved in plant immunity (Ding et al. 2024; Furuta et al. 2024; Hu et al. 2021; Liu et al. 2025a, b; Qi et al. 2015b; Song et al. 2017; Yi et al. 2020). JA-associated MYC2-like (JAMs), belonging to the IIId bHLH subclade, interact with JAZ proteins to repress JA-responsive genes and competitively bind to the G-box motif in the promoters of MYC target genes (Nakata et al. 2013; Sasaki-Sekimoto et al. 2013; Song et al. 2013). WRKY-type TFs are required for JA-mediated defense responses against RKNs in tomato (Huang et al. 2025, 2022; Zhao et al. 2023). The SlJAZ-interacting TF SlWRKY30llc cooperates with the VQ-motif-containing protein SlVQ15 to bind to the promoters of *SlJAZ* genes and repress their expression, thereby enhancing tomato resistance to the RKN *Meloidogyne incognita* (Huang et al. 2025). By contrast, SlWRKY45 interacts with many SlJAZ proteins, represses the expression of the JA biosynthesis gene *SlAOC*, and thereby decreases tomato resistance to RKNs (Huang et al. 2025).

In addition to their well-documented roles in biotic stress tolerance, specific JAZ–TF modules also regulate plant responses to abiotic stresses. Cold stress induces the accumulation of JA, leading to JA-mediated degradation of JAZ proteins. JAZ degradation releases the transcriptional repression of subgroup IIIb bHLH proteins, specifically the TFs inducer of CBF expression 1 (ICE1) and ICE2, which are key regulators of cold responses. Consequently, ICE TFs activate the expression of *C-repeat binding factor/DRE binding factor 1* (*CBF/DREB1*) genes and the cold-responsive genes that are regulated by their encoded proteins (Hu et al. 2013b). In rice, JAZ–bHLH modules also contribute to salinity tolerance. OsJAZ9 and OsbHLH062 interact to modulate ion homeostasis, and rice salt-sensitive3 (RSS3) functions as an adaptor protein to link JAZ with bHLH proteins in a ternary complex that regulates root growth under high-salt conditions (Toda et al. 2013). In addition, JAZ proteins modulate salinity tolerance by interacting with nuclear factor-Y (NF-Y) subunits. An NF-Y complex, composed of NF-YA1, NF-YB2, and NF-YC9, positively regulates the expression of salinity-responsive genes. However, JAZ8 directly interacts with these subunits, inhibiting both the formation of the NF-YA1–YB2–YC9 ternary complex and its transcriptional activity (Li et al. 2024c). Phosphate (Pi) deficiency triggers JA-responsive gene expression, and this response requires COI1 function. Recent research has demonstrated that JAZs physically interact with Pi signaling-related MYB TFs that have a MYB domain and a coiled-coil domain (MYB-CCs), such as phosphate starvation response 1 (PHR1) and its homologs. JAZ degradation promotes the interaction of MYC2 with PHR, which leads to JA-mediated suppression of root growth and promotion of anthocyanin accumulation (He et al. 2023).

These JAZ–TF modules also regulate plant development. Arabidopsis JAZ proteins bind MYCs and MYBs (MYB21 and MYB24), forming a JAZ–bHLH–MYB ternary complex that regulates stamen and pollen production (Huang et al. 2017a; Qi et al. 2015a; Song et al. 2011). In tomato, SlJAZ9 interacts with the flower-specific MYB TF SlMYB21 to control ovule development (Schubert et al. 2019). Jasmonates interact with the APETALA 2 (AP2) TFs TARGET OF EAT1 (TOE1) and TOE2, which modulate the expression of *FLOWERING LOCUS T*, thus delaying flowering (Zhai et al. 2015). JA plays a central role in trichome development by modulating transcription factor activity. In addition, JAZ proteins modulate trichome initiation and anthocyanin biosynthesis through direct interactions with MYB–bHLH–WD-repeat (MBW) complexes; MBW complexes include the WD-repeat protein transparent testa glabra1 (TTG1), members of subgroup IIIf bHLH TFs transparent testa 8 (TT8), enhancer of glabra 3 (EGL3), glabra 3 (GL3), and the R2R3 MYB TFs MYB75 and glabra 1 (GL1) (Qi et al. 2011). In tomato, SlJAZs interact with various TFs, including the homeodomain–leucine zipper (HD-ZIP) homeodomain protein 8 (HD8) and the SlWoolly–SlMYC1 regulatory module (Hua et al. 2021a, b). JA promotes leaf senescence across various plant species (Hu et al. 2017). JAZ proteins interact with YABBY TFs, such as FILAMENTOUS FLOWER (FIL) and YAB3; this interaction contributes to JA-mediated chlorophyll degradation, anthocyanin accumulation, and disease resistance (Boter et al. 2015). Furthermore, JAZ4, JAZ8, and the auxin signaling repressor indole-3-acetic acid inducible 29 (IAA29) competitively interact with WRKY57 in Arabidopsis, thereby exerting antagonistic effects on JA-induced leaf senescence (Jiang et al. 2014). JAZ proteins physically interact with the group VIII bHLH TFs root hair defective 6 (RHD6) and RHD6-like 1 (RSL1), and disrupt the RHD6–RSL1 interaction, thereby modulating development of root hairs (Han et al. 2020). Recently, the JA-induced ethylene response factors ERF109, ERF114, and ERF115 were reported to interact with JAZ8, disrupting MYC2/RHD6–JAZ8 complex formation to promote root hair development (Sui et al. 2025). JA signaling also participates in the light-signaling pathway to regulate cell elongation and seedling morphogenesis. JAZ3 regulates photomorphogenesis and thermomorphogenesis by inhibiting the transcriptional regulation of the bHLH TF phytochrome-interacting factor 4 (PIF4), a key integrator of light and temperature signals in seedling morphogenesis (Huai et al. 2024). Furthermore, squamosa promoter binding-like (SPL) TFs interact with JAZ3 to promote its accumulation, modulating age-dependent JA-mediated defense responses (Mao et al. 2017).

JAZ–TF modules facilitate extensive interactions between the signaling pathways of JA and other phytohormones, thereby coordinating plant growth and defense. JAZ proteins contribute to ethylene signaling by interacting with ethylene insensitive 3 (EIN3) and EIN3-like 1 (EIL1), which integrate JA and ethylene signaling to regulate root hair development and plant immunity against necrotrophic pathogens (Zhu et al. 2011). In addition, JA and ABA signaling intersect during seed germination and post-germinative growth. JAZs physically interact with ABA-insensitive 3 (ABI3) and ABI5, essential components of the ABA signaling pathway, to modulate transcriptional activity and coordinate JA–ABA crosstalk (Pan et al. 2020). Gibberellins (GAs) and JA synergistically regulate trichome development and filament elongation (Huang et al. 2020; Qi et al. 2014). This synergy involves GA-induced degradation of DELLA repressors and JA-induced degradation of JAZ repressors, which activates the MBW transcriptional complex and initiates trichome formation and filament elongation in a manner dependent on both JA and GA (Huang et al. 2020; Qi et al. 2014). Moreover, during herbivore attack in rice, JA signaling triggers defense responses and facilitates GA catabolism concurrently, allowing rapid resource allocation optimization via the MYC2–GA2-oxidase (GA2ox) and JAZ–DELLA modules (Jin et al. 2023).

These studies highlight the critical roles of JAZ–TF modules in regulating JA-mediated plant stress responses, growth, and development. There are 13 Arabidopsis JAZs, 12 tomato JAZs, and 15 rice JAZ proteins. Previous studies in Arabidopsis have reported specificity and redundancy among JAZ proteins using single and higher order *jaz* mutants (e.g., quintuple [lacking function for five JAZs], decuple [loss of ten JAZs], and undecuple [loss of 11 JAZs] mutants) (Guo et al. 2018b; Liu et al. 2021a). However, how these proteins specifically regulate JA-mediated plant stress responses and development has not been fully elucidated. In addition, beyond well-studied crops, such as Arabidopsis, rice, and tomato, the functional conservation and diversity of JAZ–TF modules in other species are poorly understood and merit further research.

## Attenuation of the JA signaling pathway

JA-triggered immunity is a typical inducible defense. Indeed, plants activate defense responses only when subject to biotic or abiotic stress, thereby enhancing plant fitness in an efficient and energy-conserving manner. Overactivation of JA responses can negatively affect plants, including their growth, fertility, and photosynthetic output, even leading to cell death (Baldwin 1998; Guo et al. 2018a; Yan et al. 2007; Zhang and Turner 2008). Plants have evolved a sophisticated negative feedback transcriptional regulatory network centered around MYC2. MYC2 not only activates and amplifies JA signaling but also initiates its attenuation by inducing the expression of genes encoding various negative regulators, such as the systemin receptor (*SYR2*), JA metabolic enzymes, transcriptional repressors (*JAZ/JAV1*), and competitive TFs (*JAM/MTB*) (Fig. 2B). This feedback loop precisely regulates the intensity and duration of JA-induced defense responses, providing a key example for research in plant hormone biology.

### MYC2-induced SYR2 attenuates systemin signaling

Wounding induces the expression of the negative regulator gene *SYR2*, encoding a systemin receptor, in a MYC2-dependent manner. Overexpression of *PROSYSTEMIN* activates constitutive expression of jasmonate-mediated defense genes and inhibits plant growth, suggesting that overactivation of the systemin signaling pathway impedes plant growth (McGurl et al. 1994). Recent studies have identified SYR1 as a high-affinity systemin receptor that enhances the systemin signal and systemic woundresponses, and SYR2 as a low-affinity systemin receptor that decreases systemin signaling and systemic wound responses. Wound-induced systemin accumulation promotes the interaction between SYR1 and the co-receptor SOMATIC EMBRYOGENESIS RECEPTOR KINASE 3a (SERK3a), initiating and amplifying systemic wound responses. At the same time, MYC2 directly binds to the promoters of *PROSYSTEMIN* and *SYR2*, activating their expression and leading to the production of more systemin and SYR2. Under high systemin concentrations, SYR2 outcompetes SYR1 for binding to their co-receptor SERK3a, thereby attenuating systemic wound responses (Zhou et al. 2025). This finding gives new insight into the mechanisms involved in the negative regulation of signaling pathways at the level of PRR complexes. Previous studies have reported molecular mechanisms in which antagonistic peptides competitively bind to the same receptor to optimize various aspects of plant development, including stomatal patterning, pollen tube rupture, and pollen germination (Ge et al. 2017; Lee et al. 2015; Liu et al. 2021b). Therefore, whether systemin-like antagonists exist and compete with systemin for SYR1 binding to suppress the JA signaling pathway and achieve JA homeostasis remains an open question.

### MYC2-induced JA metabolic genes promote the jasmonate catabolism

JA responses are closely linked to the levels of JA and bioactive JA-Ile. A striking feature of JA-Ile is its rapid accumulation under conditions of tissue damage or other stress stimuli, occurring within seconds to minutes (Glauser et al. 2008; Koo et al. 2009). Simultaneously, wounding induces the expression of numerous JA catabolic genes in a MYC2-dependent manner, including *CYP94B1*, *CYP94B3*, *CYP94C3*, *JMT*, *JOX*s, and *JAO*s (Chen et al. 2024; Van Moerkercke et al. 2019; Zhang et al. 2020a). The catabolism of JA-Ile or its precursor effectively terminates the JA signaling pathway. Three major pathways are known for the degradation of JA-Ile or limit its biosynthesis in the cytoplasm: (1) in the ω-oxidation pathway, CYP94 subfamily cytochrome P450 enzymes catalyze the successive oxidation of JA-Ile, producing 12-hydroxy-JA-Ile (12OH-JA-Ile) and 12-carboxy-JA-Ile (12COOH-JA-Ile) (Aubert et al. 2015; Heitz et al. 2012; Koo et al. 2014, 2011; Yang et al. 2024a); (2) in the hydrolytic pathway, aminohydrolases cleave JA-Ile to JA and Ile (Widemann et al. 2013; Woldemariam et al. 2012; Zhang et al. 2016); and (3) JA-Ile production can also be restricted by limiting the availability of its precursor JA. JA oxidases (JOX or JAO), which belong to the 2-oxoglutarate-dependent dioxygenase family, catalyze JA oxidation to 12-hydroxy-JA (12OH-JA), thereby redirecting metabolic flow away from JA-Ile biosynthesis (Caarls et al. 2017; Smirnova et al. 2017). Furthermore, JA CARBOXYL METHYLTRANSFERASE (JMT) can convert JA into the volatile MeJA, leading to lower JA-Ile accumulation (Seo et al. 2001; Stitz et al. 2011). In addition, jasmonate-induced dioxygenase 1 (JID1) is a 2-oxoglutarate/Fe(II)-dependent dioxygenase that modifies the JA precursor OPDA, thereby preventing its conversion to JA and JA-Ile. Nevertheless, the chemical structure of the modified OPDA remains unclear due to technical limitations (Yi et al. 2023).

### MYC2-induced JAZs restore transcriptional repression

JAZs act as key transcriptional repressors in JA signaling, and the expression of their encoding genes is induced early by JA and wounding in a MYC2-dependent manner (Chini et al. 2007; Thines et al. 2007). These resulting JAZs interact with TFs such as MYC2 to re-establish the repression of MYC2 function (Chini et al. 2007; Chung et al. 2008; Thines et al. 2007; Yan et al. 2007). Moreover, the stability of JAZ proteins determines the strength of their repressive activity. The mechanisms underlying the JAZ protein stability mainly include the following three aspects. (1) Non-canonical JAZ degron-containing JAZ proteins: the JAZ degron is the minimum amino acid sequence within the Jas domain of canonical JAZ proteins that can interact with JA-Ile and COI1 (Melotto et al. 2008; Sheard et al. 2010). JAZ8 and JAZ13 contain a non-canonical degron lacking the LPIARR loop region essential for JA-Ile-induced JAZ–COI1 interaction (Sheard et al. 2010; Thireault et al. 2015). Thus, they weakly interact with COI1 in a JA-Ile-dependent manner while maintaining the capacity to bind target TFs and recruit co-repressors for transcriptional repression (Shyu et al. 2012; Thireault et al. 2015). Therefore, overexpression of *JAZ8* or *JAZ13* results in lower sensitivity to JA treatment, whereas there was no significant effect of overexpression of *JAZ* genes encoding proteins with a canonical degron (Chini et al. 2007; Thines et al. 2007; Yan et al. 2007). (2) Dominant JAZ splice variants: the highly conserved Jas intron found in the majority of Arabidopsis JAZ genes undergoes alternative splicing, resulting in a set of transcripts that encode truncated JAZ proteins lacking a complete C-terminal Jas domain (Chung et al. 2010; Chung and Howe 2009; Moreno et al. 2013; Wu et al. 2020; Yan et al. 2007). Overexpressing these truncated *JAZ* splice variants diminishes JA responses, as the proteins encoded by these splice variants still interact with MYC2 and also are more resistant to JA-Ile-induced degradation than their intact counterparts (Chung et al. 2010; Chung and Howe 2009; Moreno et al. 2013). (3) Post-translational modifications: for instance, the expression of the U-box E3 ubiquitin ligase gene *SlPUB22* is significantly induced under insect attack in tomato plants, and SlPUB22 mediates the degradation of non-COI1-targeted JAZ proteins via ubiquitination, positively regulating the defense response and JA signaling pathway (Wu et al. 2024a). SKP1-interacting partner 31 (SKIP31), an abundant F-box protein in Arabidopsis seeds, promotes the degradation of JAZ6 and JAZ11 in a CO1-dependent manner, thereby relieving the repression of ABI5 and activating the expression of genes associated with seed maturation (Varshney et al. 2023). In addition, the SUMO protease OVERLY TOLERANT TO SALT 1 (OTS1) plays a key role in regulating the SUMOylation of JAZ proteins in Arabidopsis, which affects the JAZ–COI1 interaction and controls JAZ protein abundance to enable rapid biotic stress responses (Srivastava et al. 2018). OsPRMT6a (protein arginine methyltransferase 6a) catalyzes arginine methylation of OsJAZ1 and OsJAZ7, which enhances their interaction with OsCOI1 in a JA-dependent manner and then facilitates the ubiquitination and degradation of OsJAZ1. Mutation of *OsPRMT6a* promotes the stability of OsJAZ1, leading to diminished JA responses and spikelet defects, reminiscent of the phenotypes of a methylation-resistant OsJAZ1 variant and the *Oscoi1a Oscoi1b* double mutant (Dong et al. 2024).

JAV1 is a plant-specific protein containing a conserved VQ motif and negatively regulates JA-induced defense responses (Hu et al. 2013a; Yan et al. 2018a). *JAV1-*RNAi plants silenced for *JAV1* by RNA interference (RNAi) showed significantly greater resistance to *Botrytis cinerea* and *Spodoptera litura* than wild-type plants, without growth defects (Hu et al. 2013). Like *JAZ* genes, *JAV1* expression is induced early by MYC2 following JA treatment or wounding, but JAV1 protein degradation is independent of COI1, suggesting the involvement of an unknown E3 ubiquitin ligase in this regulation (Hu et al. 2013; Wu et al. 2024b). In healthy plants, the JAV1–JAZ8–WRKY51 (JJW) ternary complex binds to the W-box in the promoter region of the JA biosynthesis gene *AOS* and suppresses its expression, thereby maintaining low levels of JA. Upon insect attack, Ca^2+^-dependent JAV1 phosphorylation triggers the dissociation of the JJW complex, leading to derepression of *AOS* expression and rapid JA accumulation (Yan et al. 2018a). The MYC2 homolog Gossypium Pigment Gland Formation (GoPGF) of cotton directly activates expression of *JAVL*, a *JAV1* homolog. JAVL then represses JA biosynthesis genes and directly interacts with GoPGF to inhibit its transcriptional activity, thereby achieving JA homeostasis (Wu et al. 2024b).

### MYC2-induced JAMs compete with MYC2 for transcriptional repression

The Arabidopsis JAM family is composed of bHLH TFs closely related to and functionally antagonistic to MYC2. JAM homologs in tomato are referred to as MYC2-related transcription factor B (MTB) (Fonseca et al. 2014; Liu et al. 2019; Nakata et al. 2013; Sasaki-Sekimoto et al. 2013; Song et al. 2013). The expression of *JAM* and *MTB* genes is induced early by MYC2 in response to JA and mechanical wounding, and their protein products compete with MYC2 for binding to the G-box in the promoters of MYC2 target genes (Fonseca et al. 2014; Liu et al. 2019; Nakata et al. 2013; Sasaki-Sekimoto et al. 2013; Song et al. 2013). Nonetheless, in the absence of the conserved MYC2 activation domain, MTBs can only interact with JAZs, but not with MED25, and act solely as transcriptional repressors to regulate MYC2 target genes (Liu et al. 2019). In addition, JAMs can directly interact with JAZ proteins, imposing transcriptional repression by recruiting the co-repressors NINJA and TPL (Fonseca et al. 2014; Song et al. 2013).

### Additional negative regulation

In addition to the MYC2-mediated negative feedback mechanism, previous studies have reported that phytohormone (e.g., GA, ethylene, and salicylic acid [SA]) signaling pathways and the light signaling pathway interact antagonistically with the JA signaling pathway. JA and GA promote the proteasome-dependent degradation of JAZ and DELLA proteins, respectively, thereby relieving the inhibition of MYC TFs or PIFs involved in growth. Without GA, stabilized DELLA competes with MYC2 for binding to JAZ1, thereby relieving the inhibition of MYC2 imposed by JAZ and activating JA responses. During plant growth, GA-induced degradation of DELLA releases JAZ, facilitating the repression of MYC2 function by JAZ and consequently suppressing JA responses (Hou et al. 2010).

Ethylene-mediated negative regulation of JA responses is a key mechanism for terminating JA signaling. On one hand, the ethylene-stabilized TF EIN3 interacts with and represses MYC2, inhibiting JA-mediated defenses against pathogens and insects (Song et al. 2014). On the other hand, EIN3 and its homolog EIL1 interact with the MBW transcriptional complex to decrease anthocyanin levels, formation of trichomes, and resistance to insects (Song et al. 2022).

In general, plants defend themselves against chewing insects and necrotrophic pathogens via the JA pathway, whereas they defend against piercing-sucking insects and biotrophic pathogens via the SA pathway, with the two signaling pathways being mutually antagonistic (Spoel and Dong 2024). SA represses JA accumulation via its interaction with the H_2_O_2_-scavenging enzyme CATALASE 2 to limit the activity of the JA biosynthesis enzymes ACYL-COA oxidase 2 (ACX2) and ACX3 (Yuan et al. 2017). Moreover, the repression of several JA biosynthesis genes by SA is mediated through the SA receptor NONEXPRESSOR OF PR GENES 1 (NPR1) (Leon-Reyes et al. 2010; Spoel et al. 2003). SA-induced NPR1 interacts with nuclear MYC2 and is recruited to the promoters of MYC2 target genes, preventing Mediator complex recruitment and thus inhibiting the JA-induced activation of gene expression (Nomoto et al. 2021).

Under shade conditions, the light receptor phytochrome B (phyB) inhibits JA defense responses by regulating MYC and JAZ protein stability, thereby prioritizing elongation growth (Ballare 2014; Chico et al. 2014; Moreno et al. 2009). Recently, the IVa bHLH transcription factors bHLH19 and bHLH20, along with the IIIa bHLH factor ABORTED MICROSPORES (AMS, also known as DYSFUNCTIONAL TAPETUM 1 [DYT1]), have been identified as novel negative regulators of JA-induced insect defense. They function by interacting with components of the MYC–MYB complex and repressing its transcriptional activity (Pang et al. 2024; Song et al. 2025). Although these studies have uncovered antagonistic interactions between JA and other signaling pathways, the precise underlying regulatory mechanisms require further investigation.

## Conclusion and prospects

This review provides a systematic overview of recent advances in the molecular regulatory network of JA biosynthesis, metabolism, transport, and signaling. It focuses on the important role of the MYC2-mediated feedback loop in JA homeostasis, providing an important paradigm for understanding phytohormone regulatory mechanisms. Precise regulation of key genes within the multilayered MYC2-mediated negative feedback loops in the JA signaling pathway can enhance stress resistance without impairing plant growth, providing a strategy to overcome the growth-defense trade-off. For example, SlCYP94C1 in tomato is a cytochrome P450 enzyme that converts JA-Ile into its inactive forms. Mutation of *CYP94C1* resulted in significantly greater resistance to necrotrophic pathogens without affecting fruit ripening or key nutritional components (e.g., sugars, acids, lycopene, and vitamin C) (Yang et al. 2024a). In addition, clustered regularly interspaced short palindromic repeats (CRISPR)/CRISPR-associated nuclease 9 (Cas9)-mediated gene editing of *OsJAZ10* yielded the variant FJ10 (Frameshift mutation of OsJAZ10), which produces a variant of OsJAZ10 lacking the Jas domain and enhanced plant resistance to herbivores without yield penalty. The FJ10 version of OsJAZ10 does not interfere with canonical JA signaling. Instead, it interacts with slender rice 1 (OsSLR1) and F-box/KELCH 16 (OsFBK16), thereby attenuating the inhibition of gibberellin-mediated growth by OsSLR1 and that of lignin-based defense responses by OsFBK16 (Li et al. 2024a). Similarly, silencing Arabidopsis *JAV1* or its cotton homolog *JAVL* (*JAV1-LIKE*) enhanced plant resistance to fungi and insects and induced the accumulation and biosynthesis of defense compounds, all without compromising normal growth (Hu et al. 2013a; Wu et al. 2024b). Notably, knocking out tomato *MTB* via CRISPR/Cas9 improved resistance to insects while maintaining normal growth and development (Liu et al. 2019).

Several key scientific questions remain to be addressed. First, in terms of upstream signal perception and transduction in JA signaling, it is essential to elucidate how plants sense biotic and abiotic stresses through DAMPs, HAMPs, or physical signals, as well as to decipher the molecular mechanisms that coordinate rapid JA accumulation in response to diverse stimuli. Moreover, for the functional characterization of JA-related bioactive derivatives, particular attention should be given to COI1-independent signaling pathways involving derivatives, such as OPDA and *cis*-jasmone. In addition, recent landmark findings have established cyclic AMP (cAMP) as a key second messenger in auxin signaling, demonstrating that the auxin receptors TIR1 and Auxin F-Box proteins (AFBs) possess adenylate cyclase activity and synthesize cAMP upon auxin binding (Chen et al. 2025; Qi et al. 2022a). Given the structural analogy between COI1 and TIR1, further investigation is required to determine whether cAMP is involved in the JA signaling pathway and whether COI1 also exhibits adenylate cyclase activity. Furthermore, with regard to growth-defense trade-offs, it is imperative to elucidate how JA regulates the spatiotemporal specificity of organ development and stress responses, thereby informing strategies for the design of high-yielding and stress-resistant crops. In addition, the crosstalk mechanisms between JA and SA need to be explored, particularly how plants precisely distinguish between different pathogen and herbivore types to initiate specific defense responses. The recent achievement of de novo biosynthesis of JA-Ile and MeJA from glucose in *Saccharomyces cerevisiae* (Tang et al. 2024) provides a foundation for exploring the potential application of synthetic jasmonates and their analogs as biopesticides, which warrants further investigation. Finally, the JA signaling pathway plays an essential role in plant regeneration (Yang et al. 2024b; Zhang et al. 2023a; Zhou et al. 2019). Research into the interaction between JA and other wound-induced signals and phytohormones will advance our understanding of the complex network that governs regeneration after wounding.

## Data Availability

Data sharing not applicable to this article as no data sets were generated or analyzed during this study.
